# An optimal combination of four active components in Huangqin decoction for the synergistic sensitization of irinotecan against colorectal cancer

**DOI:** 10.1186/s13020-024-00967-1

**Published:** 2024-07-02

**Authors:** Hongyan Zhou, Dingxin Hu, Xian Zhao, Siyuan Qin, Qiyao Nong, Yuan Tian, Zunjian Zhang, Haijuan Dong, Pei Zhang, Fengguo Xu

**Affiliations:** 1 Key Laboratory of Drug Quality Control and Pharmacovigilance (Ministry of Education), State Key Laboratory of Natural Medicine, China Pharmaceutical University, Nanjing, 210009 People’s Republic of China; 2 Department of Pharmacy, China Pharmaceutical University, Nanjing Drum Tower Hospital, Nanjing, 210008 People’s Republic of China; 3 Department of Pharmacy, Affiliated Hospital of Medical School, Nanjing Drum Tower Hospital, Nanjing University, Nanjing, 210008 People’s Republic of China; 4 The Public Laboratory Platform, China Pharmaceutical University, Nanjing, 210009 People’s Republic of China; 5 School of Traditional Chinese Pharmacy, China Pharmaceutical University, Nanjing, 210009 People’s Republic of China

**Keywords:** Huangqin decoction, Colorectal cancer, Irinotecan, Orthogonal design, Synergistic effect, Fatty acid metabolism

## Abstract

**Background:**

Irinotecan (CPT-11) is a first-line treatment for advanced colorectal cancer (CRC). Four components (baicalin, baicalein, wogonin, and glycyrrhizic acid) derived from Huangqin Decoction (HQD) have been proven to enhance the anticancer activity of CPT-11 in our previous study.

**Objective:**

This study aimed to determine the optimal combination of the four components for sensitizing CPT-11 as well as to explore the underlying mechanism.

**Methods:**

The orthogonal design method was applied to obtain candidate combinations (Cmb1-9) of the four components. The influence of different combinations on the anticancer effect of CPT-11 was first evaluated in vitro by cell viability, wound healing ability, cloning formation, apoptosis, and cell cycle arrest. Then, a CRC xenograft mice model was constructed to evaluate the anticancer effect of the optimal combination in vivo. Potential mechanisms of the optimal combination exerting a sensitization effect combined with CPT-11 against CRC were analyzed by targeted metabolomics.

**Results:**

In vitro experiments determined that Cmb8 comprised of baicalin, baicalein, wogonin, and glycyrrhizic acid at the concentrations of 17 μM, 47 μM, 46.5 μM and 9.8 μM respectively was the most effective combination. Importantly, the cell viability assay showed that Cmb8 exhibited synergistic anticancer activity in combination with CPT-11. In in vivo experiments, this combination (15 mg/kg of baicalin, 24 mg/kg of baicalein, 24 mg/kg of wogonin, and 15 mg/kg of glycyrrhizic acid) also showed a synergistic anticancer effect. Meanwhile, inflammatory factors and pathological examination of the colon showed that Cmb8 could alleviate the gastrointestinal damage induced by CPT-11. Metabolic profiling of the tumors suggested that the synergistic anticancer effect of Cmb8 might be related to the regulation of fatty acid metabolism.

**Conclusion:**

The optimal combination of four components derived from HQD for the synergistic sensitization of CPT-11 against CRC was identified.

**Supplementary Information:**

The online version contains supplementary material available at 10.1186/s13020-024-00967-1.

## Introduction

Colorectal cancer (CRC) refers to malignant tumors occurring in the colon and rectum. It is associated with significant morbidity and mortality worldwide [1]. Surgical resection remains the first-line treatment of CRC, but 30 ~ 50% of the patients will experience recurrence [2]. Chemotherapy is one of the most important adjuvant therapies in clinical, among which irinotecan (CPT-11) is often used for the treatment of advanced or metastatic CRC [3]. However, limited effects on survival outcomes and severe gastrointestinal toxicity [4, 5] hamper the further application of CPT-11.

Huangqin Decoction (HQD) is a traditional Chinese medicine (TCM) used to treat nausea, vomiting, and diarrhea from Shang-Han-Lun that has been used for nearly 1800 years [6]. It is comprised of Scutellariae Radix (*Scutellaria baicalensis Georgi*, Huangqin), Paeoniae Radix Alba (*Paeonia lactiflora* Pall., Baishao), Glycyrrhizae Radix et Rhizome (*Glycyrrhiza uralensis* Fisch., Gancao), and Jujubae Fructus (*Ziziphus jujuba* Mill., Dazao). Recent studies showed that HQD could alleviate dextran sulfate sodium (DSS)-induced ulcerative colitis (UC) in mice by inhibiting inflammation [7]. It was also reported that a modified preparation of HQD (PHY906) can not only reduce the gastrointestinal toxicity caused by CPT-11 but also enhance its anticancer effect [8]. Our previous study demonstrated that four components (baicalin, baicalein, wogonin, and glycyrrhizic acid) at their original ratio in HQD can enhance the anticancer effect of CPT-11 in a CRC xenograft model and showed similar pharmacological activities with HQD [9]. However, the optimal ratio of the four components combined with CPT-11 against CRC remains elusive.

In this study, the orthogonal design was used to determine the optimal combination of the four components against CRC in vitro. We demonstrated that the optimal combination could exert synergistic anticancer activity combined with CPT-11 both in vitro and in vivo. In addition, this combination could also alleviate the gastrointestinal toxicity induced by CPT-11. The underlying mechanism of the synergistic effect was explored from the perspective of metabolic regulations.

## Materials and methods

### Drugs and reagents

*Scutellaria baicalensis Georgi* (Shanxi Province), *Paeonia lactiflflora* Pall (Anhui Province), *Glycyrrhiza uralensis* Fisch (Inner Mongolia of China), and *Ziziphus jujuba* Mill (Henan Province) were purchased from Beijing Tongrentang (Beijing, China) and were identified by an expert from Nanjing University of Chinese Medicine. Baicalin (CAS#21967-41-9), baicalein (CAS#491-67-8), wogonin (CAS#632-85-9), and glycyrrhizic acid (CAS#1405-86-3) were purchased from Chengdu Remifax Biotechnology Co, Ltd (Chengdu, China). Irinotecan and irinotecan hydrochloride were obtained from Aladdin Bio-Chem Technology Co, Ltd (Shanghai, China) and Jaripharm (Jiangsu, China), respectively. HQD was prepared referring to our previous study [10] and the procedures can be found in the Extended methods section in the *Supplementary Material*.

### Cell culture

Human colon cancer cell lines HCT116 and SW620 were purchased from Nanjing Kebai Biotechnology Co, Ltd (Jiangsu, Nanjing, China). The human leukemic monocyte cell line THP-1 was obtained from the Cell Bank of the Chinese Academy of Sciences. The cells were maintained in a 37 °C incubator with 5% (V/V) CO_2_. HCT116 and SW620 cells were cultured in DMEM supplemented with 10% fetal bovine serum (FBS) and 1% penicillin–streptomycin. THP-1 cells were cultured in RPMI 1640 medium supplemented with 10% FBS and 1% penicillin–streptomycin.

### MTT assay

Cell viability was determined using the MTT (3-[4,5-dimethylthiazol-2-yl]-2,5 diphenyl tetrazolium bromide) assay. Cells were seeded in 96-well plates at a density of 3000–5000 cells per well. After 24 h, the cells were treated with the respective drugs and further incubated for 48 h. Subsequently, 20 μL of freshly prepared MTT solution (5 mg/mL) was added to each well, and the plates were incubated for an additional 4 h. Afterward, 150 μL of DMSO was added to dissolve the formazan crystals formed by viable cells. The plates were gently shaken for 10 min, and the absorbance was measured at 570 nm using a Microplate Reader (TECAN infinite 200 Pro, Germany).

### Cell migration assay

Cells were seeded in 24-well plates at a density of approximately 5 × 10^4^ cells per well. Scratch assays were performed when the cells reached approximately 100% confluence. After 24 h of incubation with the respective drugs, images of migrating epithelial monolayers were captured using an inverted phase-contrast microscope (Nikon Eclipse Ti-U, Japan). All data were analyzed using Image J software.

### Clone formation experiment

Cells were seeded in a 6-well plate at a density of approximately 1 × 10^4^ cells per well. Once the cells adhered to the well, the medium was aspirated and replaced with a drug-containing medium. Upon the formation of visible colonies, the culture was terminated. Colonies were fixed with 4% paraformaldehyde, stained with 0.5% crystal violet, and counted using a stereomicroscope.

### Cell apoptosis assay

Cells were planted in 12-well plates at a density of approximately 3 × 10^5^ cells/well. After an overnight culture, the cells were treated with drugs and incubated for 48 h. The cells were then digested with EDTA-free trypsin, collected, washed twice with PBS, and resuspended in 500 μL of binding buffer. Annexin V-fluorescein isothiocyanate (FITC) and propidium iodide (PI) double labeling was induced at room temperature in the dark for 5–15 min. The results were analyzed immediately by a BD Accuri^™^ C6 flow cytometer (BD Biosciences, US). Each experiment was independently performed at least three times. All data were analyzed using FlowJo software.

Cells were seeded in 12-well plates at a density of approximately 3 × 10^5^ cells per well. Following an overnight culture, the cells were treated with the respective drugs and then incubated for 48 h. Subsequently, the cells were detached using EDTA-free trypsin, collected, washed twice with PBS, and resuspended in 500 μL of binding buffer. Annexin V-fluorescein isothiocyanate (FITC) and propidium iodide (PI) double labeling was initiated at room temperature in the dark for 5–15 min. The results were promptly analyzed using a BD Accuri^™^ C6 flow cytometer (BD Biosciences, USA). Each experiment was independently performed at least three times. All data were analyzed using FlowJo software.

### Cell cycle assay

Cells were seeded in 12-well plates at a density of approximately 3 × 10^5^ cells per well and cultured overnight. Following overnight culture, the cells were treated with drugs and further incubated for 48 h. After the incubation, the cells were detached, harvested, and washed with PBS. Subsequently, they were fixed with 50% methanol for 2 h. Then, 1 mL of DNA staining solution and 10 μL of permeabilization solution were added, and the cells were incubated in the dark for 30 min at room temperature. Finally, the cells were analyzed using a BD Accuri^™^ C6 flow cytometer (BD Biosciences, US), and all data were analyzed using FlowJo software.

### Enzyme-linked immunosorbent assay (ELISA)

THP-1 cells were plated in 24-well cell culture plates at a density of approximately 1 × 10^6^ cells per well. Adherent macrophages were induced by adding 100 ng/mL PMA to each well. After 48 h, the medium was replaced with fresh medium (control or model group) or medium containing drugs (treatment group). After an additional 12 h, 100 ng/mL LPS was added to the medium and incubated for 8 h. Subsequently, the medium was transferred to a 1.5 mL sterile centrifuge tube, centrifuged at 3000 × g for 10 min at 4 °C, and the supernatant was collected. The levels of inflammatory factors in the supernatant were measured according to the instructions provided with the ELISA kit.

### Colorectal cancer xenograft model

Male Balb/c athymic nude mice (5 − 6 weeks) weighing 23 ~ 25 g were obtained from Hangzhou Ziyuan Experimental Animal Technology Co, Ltd (Hangzhou, China). They were housed in a temperature-controlled environment (24 ± 2 °C) under a 12/12 h-dark/light cycle for one week to acclimatize. Following acclimatization, approximately 5 × 10^6^ HCT116 cells suspended in 0.1 mL DMEM were injected into the subcutaneous tissue. Once the tumor volume reached 400–500 mm^3^, the tumor tissue was harvested and cut into small pieces (approximately 1 mm^3^), and then transplanted subcutaneously into the armpit of each nude mouse. Upon reaching an average tumor volume (TV) of approximately 100 mm^3^, the mice were randomly divided into 6 groups (n = 8) according to the tumor size: Model, CPT-11, Cmb8, Cmb8 + CPT-11, Cmb3 + CPT-11, and HQD + CPT-11. HQD, Cmb8, and Cmb3 were suspended in distilled water. CPT-11 at a dosage of 40 mg/kg were intraperitoneally injected weekly. Cmb8 (15 mg/kg of baicalin, 24 mg/kg of baicalein, 24 mg/kg of wogonin, and 15 mg/kg of glycyrrhizic acid), Cmb3 (340 mg/kg of baicalin, 10 mg/kg of baicalein, 2.5 mg/kg of wogonin, and 15 mg/kg of glycyrrhizic acid), or HQD (10 g/kg) were administered orally every day. Body weight and TV (TV = length × width^2^/2) were monitored daily. Upon reaching an average TV of 1000 mm^3^, the animals were sacrificed. Blood and tumor samples were collected for further experiments. All the procedures were approved by the Animal Ethics Committee of China Pharmaceutical University and conducted by the Guide for the Care and Use of Laboratory Animals (No. 2022-05-047).

### Hematoxylin and eosin (H&E) staining and immunohistochemical (IHC) analysis

The fixed colon and tumor tissues were embedded in paraffin and stained using the H&E method. The expression of Ki67 in tumors was detected using the IHC method. Both IHC and H&E examinations were conducted by Wuhan Seville Biotechnology Co, Ltd (Wuhan, China).

### Chemical derivatization-based targeted metabolomics analysis

In this study, a targeted metabolomics method covering 151 metabolites, as recently published by our group [11], was employed to investigate metabolic changes following drug treatment. Frozen tumor samples (~ 30 mg) were placed into pre-cooled homogenization tubes, to which extraction solvent mixture ethanol-acetonitrile (1:1, v/v) was added at a ratio of 30 μL per milligram of tissue. The samples were then homogenized and centrifuged (14000 × g, 10 min, 4 °C). The resulting supernatant was collected, aliquoted, dried, and stored at −80 °C before chemical derivatization. Detailed procedures for chemical derivatization can be found in the Extended methods section in the *Supplementary Material*.

Metabolomic analysis was conducted using a Shimadzu Nexera UPLC system interfaced with an 8060 triple quadruple mass spectrometer (Shimadzu, Kyoto, Japan) equipped with an electrospray ionization source. Details for instrument conditions, column, mobile phase, and MS parameters are provided in the recent publication [11] as well as in the Extended methods section in the *Supplementary Material*.

### Statistical analysis

All data were analyzed using GraphPad Prism 8.0 (GraphPad Software Inc., San Diego, CA). A two-tailed unpaired t-test was employed for comparing two groups, while one-way ANOVA with Tukey's test was utilized for comparing three or more groups to assess significance. Differences were considered statistically significant at p < 0.05. The synergism between CPT-11 and different combinations was evaluated by the combination index Q [12]. The Q value was calculated by the formula of $\text{Q}=\frac{{E}_{CPT-11+Cmb8}}{{E}_{CPT-11}+{E}_{Cmb8}-{E}_{CPT-11}\times {E}_{Cmb8}}$, where *E* represents the inhibitory rate. The synergy effect is defined as follows: when Q < 0.85, the two drugs are considered to have an antagonistic effect; when 0.85 ≤ Q < 1.15, the two drugs are considered to have an additive effect; and when Q ≥ 1.15, the two drugs are considered to have a synergistic effect Table 1.

**Table 1:** Four-factor (component) and three-level (concentration) orthogonal design

Level	Baicalin (μM)	Baicalein (μM)	Wogonin (μM)	Glycyrrhizic acid (μM)
1	411	117.5	46.5	980
2	86	47	15	98
3	17	19.5	4.8	9.8

## Results and discussion

### Orthogonal experiment design

Studies have indicated that HQD as an adjunct to chemotherapy significantly improves survival and enhances tumor response [13]. In our previous research, we demonstrated that four components (i.e., baicalin, baicalein, wogonin, and glycyrrhizic acid) derived from HQD exert similar anticancer effects to HQD [9]. In this study, we employed an orthogonal design to determine the optimal combination of these four components. The selection of the concentration of each compound in the orthogonal design is based on their half-maximal inhibitory concentrations (IC50) on HCT116 cells and their content in HQD. The IC50 values for baicalin, baicalein, wogonin, and glycyrrhizic acid were determined to be 86 μM, 47 μM, 15 μM, and > 500 μM respectively. The measured concentrations were 411 μM for baicalin, 19.5 μM for baicalein, 4.8 μM for wogonin, and 9.8 μM for glycyrrhizic acid in HQD (200 μg/ml). Comparing these concentrations with their respective IC50 values, if a measured concentration was larger than its IC50 value it was considered as the highest level. If it was smaller than its IC50 value it was set as the lowest level. The remaining concentration levels were adjusted by magnification or scaling down according to the ratio between the measured concentration and its IC50 value. For glycyrrhizic acid specifically, as its estimated IC50 value was greater than 500 μM but its concentration in HQD was measured at only 9.8 μΜ (lowest level), a magnification factor of 10 × was applied to determine higher levels.

The arrangement of the orthogonal experiment is shown in Table 2. In total, nine combinations (Cmb1-9) were included, with Cmb3 representing the original combination of the four components in HQD. Commonly used ratio optimization design methods include orthogonal design, uniform design, causal relationship discovery design, polarity segmentation screening design, orthogonal uniform joint design, response surface design, etc. Orthogonal design is an optimization method that uses orthogonal tables to study and deal with multi-factor experiments. These tables are characterized by balanced dispersion and systematic comparability, ensuring even distribution of test conditions with complete matching. This minimizes interference from other factors, thus providing highly representative and comprehensive insights into the selected area, facilitating effective comparisons.

**Table 2:** Arrangement of orthogonal experiments

Group	Baicalin	Baicalein	Wogonin	Glycyrrhizic acid
Cmb1	1	1	1	1
Cmb2	1	2	2	2
Cmb3	1	3	3	3
Cmb4	2	1	2	3
Cmb5	2	2	3	1
Cmb6	2	3	1	2
Cmb7	3	1	3	2
Cmb8	3	2	1	3
Cmb9	3	3	2	1

### Cmb8 showed a superior anticancer effect

**Fig. 1:**
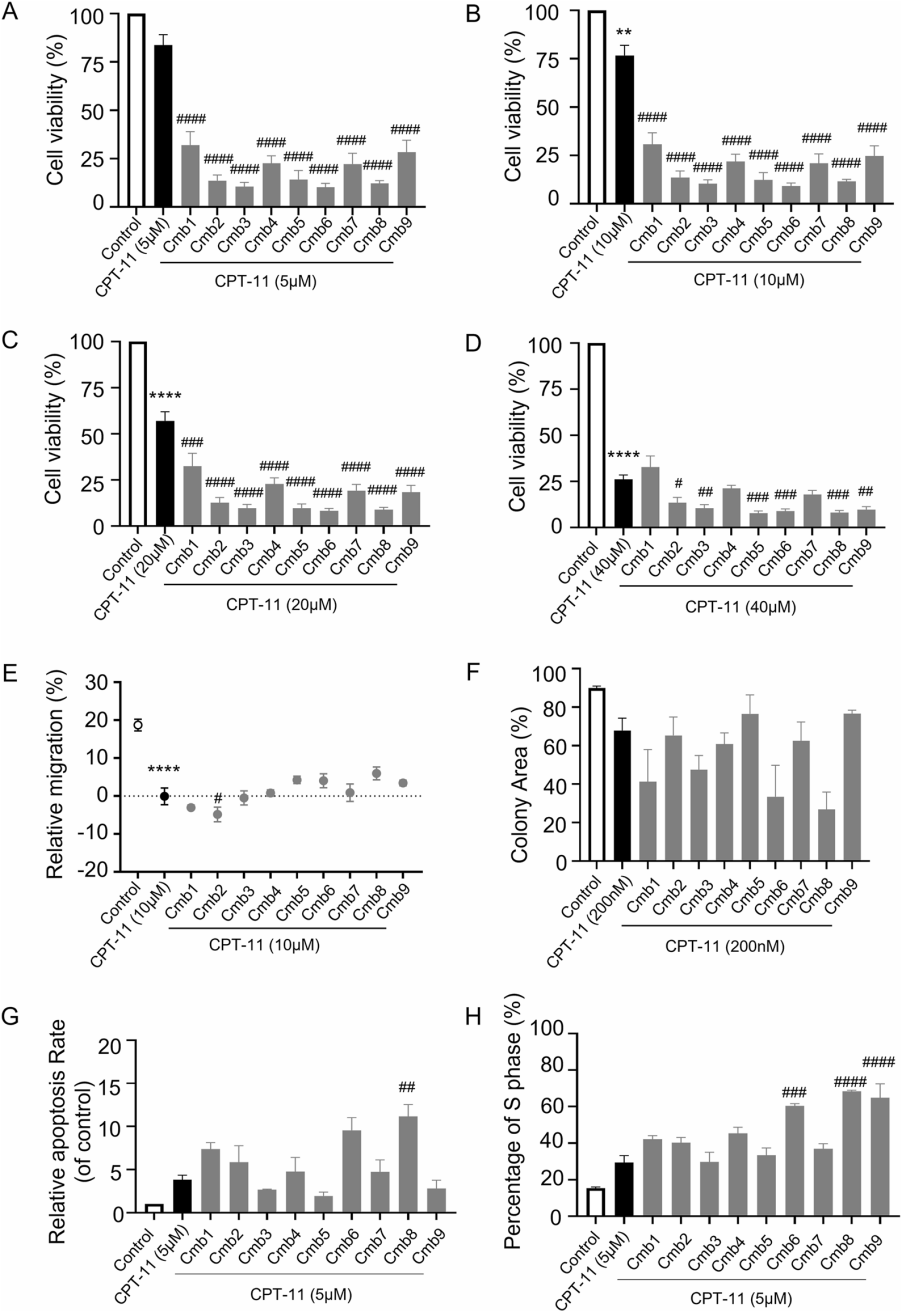
Cmb8 showed a superior anticancer effect in vitro compared to other combinations on HCT116 cells. (**A**–**D**) cell viability assay on cells treated with Cmb1-9 combined with 5, 10, 20, or 40 μM of CPT-11; **E** cell migration assay on cells treated with Cmb1-9 combined with 10 μM of CPT-11; **F** clone formation assay on cells treated with Cmb1-9 combined with 200 nM of CPT-11; **G** cell apoptosis analysis on cells treated with Cmb1-9 combined with 5 μM of CPT-11; and (**H**) cell cycle assay on cells treated with Cmb1-9 combined with 5 μM of CPT-11. One-way ANOVA with Tukey test, ^*^p < 0.05, ^**^p < 0.01, ^***^p < 0.001, ^****^p < 0.0001 in comparison with Control; ^#^p < 0.05, ^##^p < 0.01, ^###^p < 0.001, ^####^p < 0.0001 in comparison with CPT-11

We first estimated the sensitization effect of the 9 combinations on HCT116 combined with CPT-11 by cell viability assay. Following 48 h of stimulation, we observed that the cell inhibitory effect of CPT-11 at concentrations of 5, 10, and 20 μM was enhanced by Cmb1-9. Only Cmb2, 3, 5, 6, 8, and 9 exhibited a sensitization effect when CPT-11 was administered at 40 μM (Fig. 1A–D). Furthermore, the wound healing assay indicated that only Cmb2 promoted the wound healing when combined with 10 μM CPT-11 (Fig. 1E). In the clone formation experiment, we found that Cmb8 significantly contributed to the inhibitory effect of 200 nM CPT-11 (Fig. 1F). The apoptosis experiment showed that Cmb1, 6, and 8 significantly enhanced the cell apoptosis combined with CPT-11 (Fig. 1G). Additionally, Cmb1, 4, 6, 8, and 9 enhanced the effect of CPT-11 on blocking the S phase of the cell cycle, thereby inhibiting cell growth (Fig. 1H). The original images of wound healing assay, clone formation experiment, apoptosis experiment, as well as cell cycle assay are provided in Figure S1. The anticancer effect of CPT-11 and each combination alone was also evaluated and results are shown in Figure S2-S3. Upon analyzing the significance of each experiment, we observed that Cmb8 (17 μM of baicalin, 47 μM of baicalein, 46.5 μM of wogonin, and 9.8 μM of glycyrrhizic acid) exhibited a superior anticancer effect among all the combinations.

### Cmb8 showed a synergistic effect in combination with CPT-11 in vitro

**Fig. 2:**
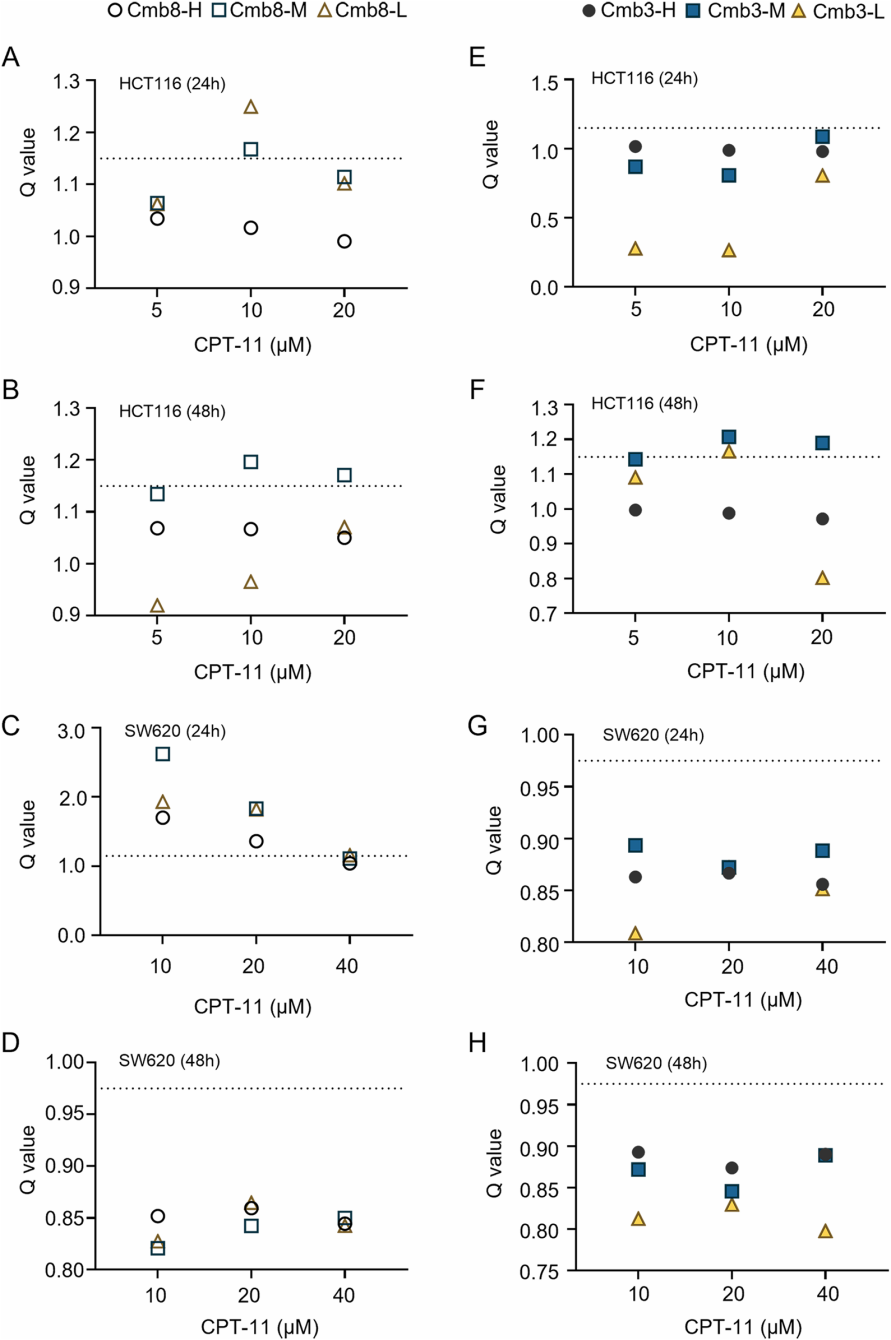
Cmb8 showed a synergistic effect in combination with CPT-11 in vitro. The combination index Q was calculated from the cell viability assays of (**A**–**D**) Cmb8 and (**E**–**H**) Cmb3 combined with different concentrations of CPT-11 treating HCT116 or SW620 cells at 24 or 48 h

Based on the above results, we evaluated the synergistic effect of Cmb8 combined with CPT-11 and compared its activity with Cmb3 which represents the original ratio of the four components (411 μM of baicalin, 19.5 μM of baicalein, 4.8 μM of wogonin, and 9.8 μM of glycyrrhizic acid) in HQD. Cell viability assays at 24 h and 48 h were conducted on both HCT116 (Figure S4) and SW620 (Figure S5) cell lines. The Q values of Cmb8 at high (Cmb8-H, 17 μM of baicalin, 47 μM of baicalein, 46.5 μM of wogonin and 9.8 μM of glycyrrhizic acid), middle (Cmb8-M, 1/2 of Cmb8-H), and low (Cmb8-L, 1/4 of Cmb8-H) concentrations combined with different levels of CPT-11 on both HCT116 (Fig. 2A-B) and SW620 (Fig. 2C–D) cells were calculated. In parallel, the Q values of Cmb3 at high (Cmb3-H, 411 μM of baicalin, 19.5 μM of baicalein, 4.8 μM of wogonin and 9.8 μM of glycyrrhizic acid), middle (Cmb3-M, 1/2 of Cmb3-H), and low (Cmb3-L, 1/4 of Cmb3-H) concentrations combined with different levels of CPT-11 on both HCT116 (Fig. 2E, F) and SW620 (Fig. 2G, H) cell lines were obtained. As evident from the results, Cmb8 demonstrated a stronger synergistic effect when combined with CPT-11 compared to Cmb3.

### Cmb8 synergistically enhanced the anti-tumor effect of CPT-11 in vivo

Based on the in vitro results, we further investigated the anticancer effect of Cmb8, Cmb3, and HQD in combination with CPT-11 in vivo. In our previous studies HQD dosage of 10 g/kg was demonstrated to exert a sensitization effect when combined with CPT-11. Based on this dosage and the component contents measured in HQD (411 μM for baicalin, 19.5 μM for baicalein, 4.8 μM for wogonin, and 9.8 μM for glycyrrhizic acid in 200 μg/ml HQD), we established the dosage of Cmb3, which reflects the original ratio of components in HQD. Then, according to the ratios of each component between Cmb3 and Cmb8 in the in vitro experiments, we further calculated the dosage of Cmb8 for the animal experiments. This calculation ensured that the dosages used in vivo were consistent with the effective concentrations identified in vitro.

**Fig. 3:**
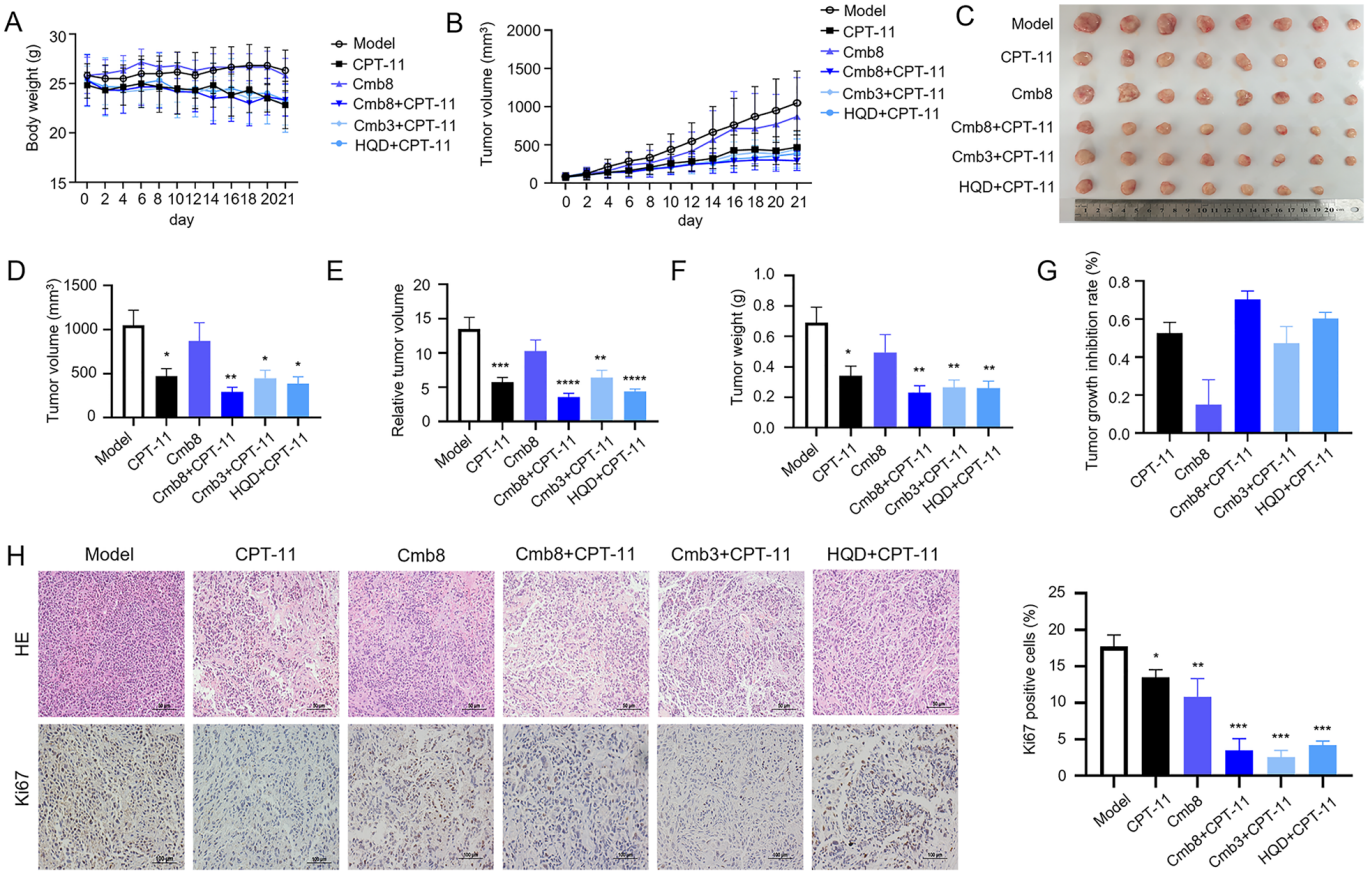
Cmb8 synergistically enhanced the anticancer effect of CPT-11 in vivo. **A** body weight of mice recorded every day; **B** tumor volume monitored over the whole experimental period; **C** images of tumors isolated from mice on day 21; **D** tumor volume on day 21; **E** relative tumor volume on day 21; **F** tumor weight on day 21; **G** tumor growth inhibition rate on day 21; **H** HE staining and Ki67 immunohistochemical examination results (400 ×). One-way ANOVA with Tukey test, ^*^p < 0.05, ^**^p < 0.01, ^***^p < 0.001, ^****^p < 0.0001 in comparison with Model

The body weight of the mice in the Model and Cmb8 groups were similar (Fig. 3A), indicating that Cmb8 alone had minimal impact on the body weight of the mice. Regarding tumor growth, as depicted in Fig. 3B–G, CPT-11, Cmb8 + CPT-11, Cmb3 + CPT-11, and HQD + CPT-11 significantly inhibited tumor growth compared to the Model group. Furthermore, Cmb8 + CPT-11 and HQD + CPT-11 demonstrated superior tumor inhibition compared to CPT-11 alone. Notably, Cmb8 synergistically enhanced the anti-tumor effect of CPT-11, with a combination index Q = 1.18, as calculated in Table 3. Histopathological sections of tumors revealed that compared with CPT-11 group, the cell apoptosis was significantly enhanced in the combinational treatment groups, particularly in Cmb8 + CPT-11 and Cmb3 + CPT-11 groups (Fig. 3H). This observation is further supported by the expression of the cell proliferation marker Ki67 in individual groups (Fig. 3H). Notably, the compounds in Cmb8 might undergo extensive metabolism in vivo, which could result in different pharmacokinetic and pharmacodynamic profiles compared to in vitro conditions. This is an important factor to consider in future investigations, as the metabolites of these compounds might contribute to or alter the observed therapeutic effects.

**Table 3:** The inhibitory rate and Q value

Group	Inhibitory rate (%)	Q value
CPT-11	52.57 ± 13.94	N.A
HQD + CPT-11	60.20 ± 8.15	N.A
Cmb3 + CPT-11	47.23 ± 19.42	N.A
Cmb8	14.96 ± 12.84	N.A
Cmb8 + CPT-11	70.42 ± 10.23	1.18

Not applicable

### Cmb8 showed an anti-inflammatory effect in vitro

**Fig. 4:**
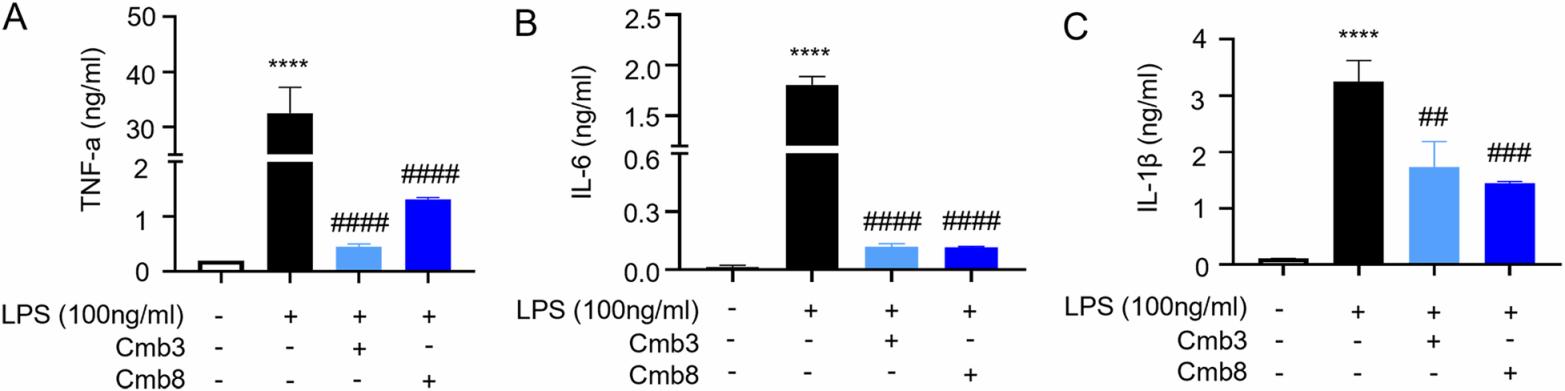
Cmb8 showed anti-inflammatory effect in vitro. **A**–**C** TNF-α, IL-6, and IL-1β levels measured on THP-1 cells treating with Cmb3 or Cmb8 after LPS stimulation. One-way ANOVA with Tukey test, ^****^p < 0.0001 in comparison with Control (no LPS); ^##^p < 0.01, ^###^p < 0.001, ^####^p < 0.0001 in comparison with Model (LPS stimulation)

Intestinal inflammation is one of the important manifestations of CPT-11-induced late diarrhea. To explore whether Cmb8 or Cmb3 could potentially mitigate the side effects of CPT-11, specifically intestinal inflammation, we assessed inflammatory factors, including TNF-α, IL-6, and IL-1β, using ELISA on THP-1 cells treated with LPS and either Cmb3 or Cmb8. The findings indicated that both Cmb8 and Cmb3 markedly decreased the levels of inflammatory factors (Fig. 4A–C).

### Cmb8 alleviated CPT-11-induced gastrointestinal toxicity in vivo

**Fig. 5:**
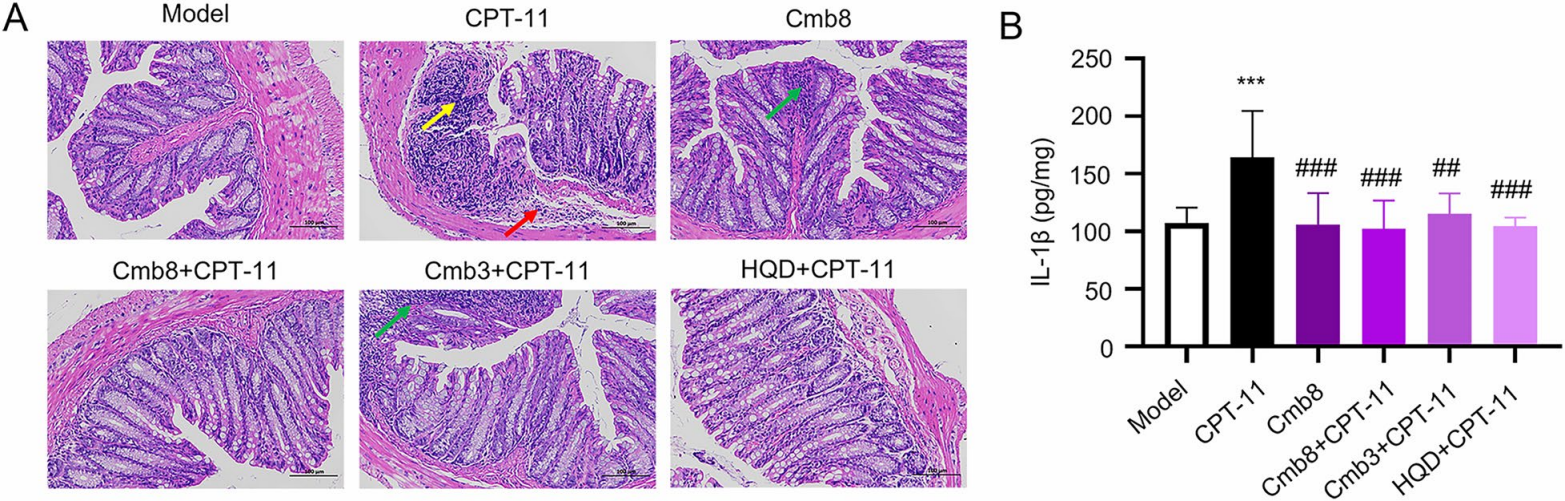
Cmb8 alleviated CPT-11-induced gastrointestinal toxicity in vivo. **A** H&E staining of the colon in different groups (200 ×); **B** Inflammatory factor IL-1β measured in the colon of mice. One-way ANOVA with Tukey test, ^***^p < 0.001 in comparison with Model; ^##^p < 0.01, ^###^p < 0.001 in comparison with CPT-11

As mentioned above, CPT-11 could induce severe gastrointestinal toxicity, especially delayed diarrhea, which limits the clinical application of the drug [5]. Here, we evaluated the protective effect of Cmb8 in alleviating CPT-11-induced adverse effects. H&E staining of the colon tissue shows that CPT-11 induced focal ulcers, mucosal epithelium sloughing, necrosis, nuclear fragmentation (yellow arrow), submucosal invasion, and an infiltrate of lymphocytes and neutrophils (red arrow) (Fig. 5A). Intramucosal lymph nodes were observed in Cmb8 and Cmb3 + CPT-11 groups (green arrow). The intestinal tissue structure in the Cmb8 + CPT-11 and HQD + CPT-11 groups appeared relatively intact, with no obvious inflammatory cell infiltration. In addition, as shown in Fig. 5B, Cmb8, Cmb3 or HQD in combination with CPT-11 significantly decreased IL-1β level in the intestines. Thus, it can be concluded that Cmb8 alleviated the intestinal damage induced by CPT-11.

### The metabolic regulations of Cmb8 in combination with CPT-11 involved fatty acid metabolism

**Fig. 6:**
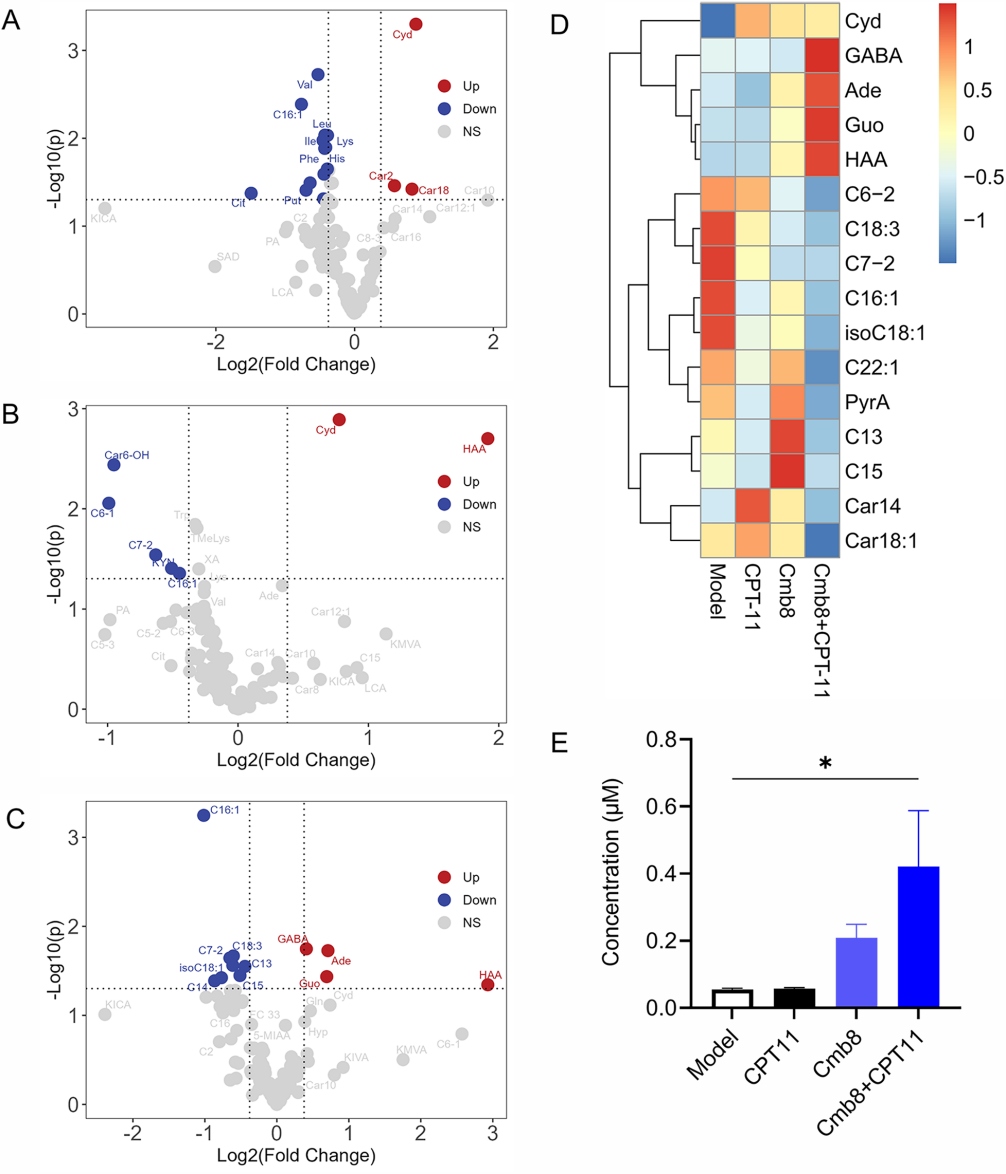
The metabolic regulations of Cmb8 in combination with CPT-11. The volcano plots showing differential metabolites in (**A**) CPT-11, **B** Cmb8, and **C** Cmb8 + CPT-11 group respectively compared to Model group; **D** Heatmap of 16 differential metabolites; **E** Histograms of HAA in different groups. One-way ANOVA with Tukey test, ^*^p < 0.05, ^**^p < 0.01. HAA, 3-hydroxybenzoic acid; Cyd, cytidine; C15, pentadecanoic acid; C13, tridecanoic acid; C7-2, 2-methylhexanoic acid; C18:3, γ-Linolenic acid; isoC18:1, elaidic acid; C16:1, palmitoleic acid; GABA, 4-Aminobutyric acid; Ade, Adenine; Guo, Guanosine; C6-2, 3-Methylvaleric acid; C22:1, Erucic acid; PyrA, Pyruvic acid; Car14, Myristoyl-L-carnitine; Car18:1, Oleoyl-L-carnitine

To investigate the underlying mechanism of the synergistic anticancer effect of Cmb8, we employed a targeted metabolomics method [11] covering 151 metabolites to study metabolic differences among the Model, Cmb8, CPT-11, and Cmb8 + CPT-11 groups. A total of 44 differential metabolites were obtained by comparing each group with the Model group using the criteria of fold change > 1.3 and P < 0.05 (Fig. 6A–C). Subsequently, we excluded metabolites showing the opposite change trends in Model, CPT-11, and Cmb8 + CPT-11 groups This filtering process resulted in the identification of 16 metabolites, and their relative concentrations were depicted in a heatmap (Fig. 6D). As we can see from the heatmap, all differential fatty acids and acylcarnitines are down-regulated in the Cmb8 + CPT-11 group compared to the Model or CPT-11 group. Furthermore, we observed a significant increase in 3-Hydroxyanthranilic acid (HAA, Fig. 6E) concentration in the Cmb8 + CPT-11 group compared to the CPT-11 group, with a sevenfold increase. Studies have indicated that HAA can inhibit the growth of sorafenib-resistant HCC cells, regulate phosphatase activity, inhibit AKT and Wnt signaling pathways, and promote the apoptosis of liver cancer cells [14, 15]. Thus, we speculate that the elevated concentration of HAA and nucleosides in the Cmb8 + CPT-11 group may contribute to its anticancer effect.

Clinical studies have reported elevated levels of fatty acids, including acetic acid, valeric acid, isobutyric acid, and isovaleric acid in CRC patients compared to healthy individuals [16]. In this study, CPT-11 decreased fatty acid levels in tumors compared to the Model group. Although there is no study reporting the regulation of CPT-11 on fatty acid metabolism, the four components in Cmb8 have been reported to regulate fatty acid metabolism via various mechanisms: (1) Baicalin can enhance the activity of carnitine palmitoyltransferase 1 (CPT1), facilitating the transport of fatty acids into mitochondria for β-oxidation [17, 18]. Baicalin reduces hepatic lipid accumulation, enhanced the phosphorylation of AMPK and ACC and down-regulated genes involved in lipogenesis, including fatty acid synthase and its upstream regulator SREBP-1c [19]. (2) Baicalein inhibits the synthesis of fatty acids through downregulation of key enzymes involved in lipogenesis, such as fatty acid synthase (FAS) and ACC. Baicalein can also promote FAO through activating AMPK and PPARα [20]. (3) Wogonin activates PPARα, leading to the upregulation of genes involved in FAO, thus enhancing the breakdown of fatty acids [21]. It inhibits SREBP-1c, leading to a decrease in the synthesis of fatty acids and triglycerides [22]. Wogonin also activates AMPK and inhibits anabolic processes such as fatty acid synthesis, helping to reduce lipid accumulation [23]. (4) Glycyrrhizic acid suppresses the expression of key lipogenic enzymes, such as FAS, reducing the synthesis of fatty acids in the liver and thereby decreasing lipid accumulation [24]. Glycyrrhizic acid promotes FAO by upregulating the expression of PPARα and its target genes, such as carnitine palmitoyltransferase 1 (CPT1) [25]. Hence, we speculate that Cmb8 may exert a synergistic anti-CRC effect when combined with CPT-11 by modulating fatty acid metabolism in tumors. Further investigations are warranted to validate these underlying metabolic mechanisms mediating the synergistic anticancer effect of Cmb8 in combination with CPT-11.

## Conclusion

In this study, we aimed to identify the optimal combination of four components (baicalin, baicalein, wogonin, and glycyrrhizic acid) derived from HQD for sensitizing CPT-11 against CRC. Our findings revealed that Cmb8 exhibited a superior anticancer effect and could synergize with CPT-11 in vitro. This synergistic effect was further confirmed in in vivo experiments. Furthermore, both in vitro and in vivo experiments demonstrated that Cmb8 could mitigate the gastrointestinal damage induced by CPT-11. Metabolic profiling of the tumors suggested that the synergistic anticancer effect of Cmb8 might be attributed to its regulation of fatty acid metabolism in tumors.

### Supplementary Information

Additional file 1.

## Data Availability

The data that support the findings of this study are available from the corresponding author upon reasonable request.

## Abbreviations

Cmb: Component combination
HQD: Huangqin decoction
CRC: Colorectal cancer
CPT-11: Irinotecan
TCM: Traditional Chinese medicine
DSS: Dextran sulfate sodium
UC: Ulcerative colitis
MTT: 3-[4,5-Dimethylthiazol-2-yl]-2,5 diphenyl tetrazolium bromide
DMSO: Dimethyl sulfoxide
PBS: Phosphate buffered saline
FITC: Fluorescein isothiocyanate
PI: Propidium iodide
DNA: Deoxyribonucleic acid
ELISA: Enzyme linked immunosorbent assay
PMA: Phorbol 12-myristate 13-acetate
LPS: Lipopolysaccharide
TV: Tumor volume
H&E: Hematoxylin–eosin
IHC: Immunohistochemical
ANOVA: Analysis of variance
IL-6: Interleukin-6
IL-1β: Interleukin-1β
TNF-α: Tumor necrosis factor-α
HAA: 3-Hydroxybenzoic acid
Cyd: Cytidine
C15: Pentadecanoic acid
C13: Tridecanoic acid
C7-2: 2-Methylhexanoic acid
C18:3: γ-Linolenic acid
isoC18:1: Elaidic acid
C16:1: Palmitoleic acid
GABA: 4-Aminobutyric acid
Ade: Adenine
Guo: Guanosine
C6-2: 3-Methylvaleric acid
C22:1: Erucic acid
PyrA: Pyruvic acid
Car14: Myristoyl-L-carnitine
Car18:1: Oleoyl-L-carnitine
