# Supplementary material for: An optimal combination of four active components in Huangqin decoction for the synergistic sensitization of irinotecan against colorectal cancer
Source: Chin Med. 2024 Jul 2;19:94. doi: 10.1186/s13020-024-00967-1 (PMC11218176; doi:10.1186/s13020-024-00967-1)
Supplement: Supplementary file 1 — Additional file 1. [file 13020_2024_967_MOESM1_ESM.docx]

*Supplementary Material*

**An optimal combination of four active components in Huangqin Decoction for the** **synergistic sensitization of irinotecan against colorectal cancer**

Hongyan Zhou ^a,#^, Dingxin Hu ^a,#^, Xian Zhao ^b,c^, Siyuan Qin ^a^, Qiyao Nong ^a^, Yuan Tian ^a^, Zunjian Zhang ^a^, [Haijuan Dong](https://pubmed.ncbi.nlm.nih.gov/?sort=pubdate&term=Dong+H&cauthor_id=37455081) ^d^, Pei Zhang ^a,⁎^, Fengguo Xu^a,e,⁎^

^a^ Key Laboratory of Drug Quality Control and Pharmacovigilance (Ministry of Education), State Key Laboratory of Natural Medicine, China Pharmaceutical University, Nanjing 210009, P. R. China

^b^ Department of Pharmacy, China Pharmaceutical University Nanjing Drum Tower Hospital, Nanjing, 210008, P.R. China

^c^ Department of Pharmacy, Nanjing Drum Tower Hospital, Aﬃliated Hospital of Medical School, Nanjing University, Nanjing 210008, P.R. China

^d^ The Public Laboratory Platform, China Pharmaceutical University, Nanjing, 210009, PR China

^e^ School of Traditional Chinese Pharmacy, China Pharmaceutical University, Nanjing 210009, P. R. China

^#^ These authors contributed equally to this work.

* Correspondence to be addressed to:

Xu FG (Tel/Fax: +86-25-83271021; Email: fengguoxu@cpu.edu.cn)

Zhang P (Tel/Fax: +86-25-83271021; Email: peizhangcpu@cpu.edu.cn)

**Extended methods**

1. The preparation of HQD

Huangqin Decoction was prepared by Scutellariae Radix (9 g), Paeoniae Radix Alba (6 g), Glycyrrhizae Radix et Rhizome (6 g), and Jujubae Fructus (6 g). These herbs were soaked in water for 30 min and decocted twice in 15 volumes and 10 volumes of distilled water (v/w) for 1.5 h and 1 h, respectively, and the decoctions were mixed. Then the merged decoctions were to dried by a freeze dry system (Labconco, USA) and stored at -80 °C until use.

2. Chemical Derivatization

For amino and phenol submetabolome derivatization, 20 μL of acetonitrile−water (1:1, v/v), 100 μL of carbonate buffer (pH 10), and 100 μL of dansyl chloride (Dns-Cl) solution (20 mM) were pipetted into each dried sample. The resulting mixture was incubated at 35°C for 15 min. After that, 40 μL of NaOH solution (100 mM) was added to the sample and incubated at 35°C for 10 min to quench the reaction. The excessive alkaline was removed by adding 40 μL of formic acid (425 mM).

For carboxyl submetabolome derivatization, the dried sample was mixed with 20 μL of acetonitrile−methanol−water (2:4:4, v/v/v), 40 μL of methanol, 20 μL of HATU solution (6 mM), and 20 μL of 5-(dimethylamino) naphthalene-1-sulfonyl piperazine (Dns-PP) solution (12 mM). After mixing, the derivatization reaction solution was incubated at 55 °C for 20 min.

Twin derivatization reagents were applied to provide one-to-one isotope derivatization-internal standards (ID-ISs). The preparation procedures of IDI-Ss were similar to the actual sample, except that the biological samples were substituted with standard mixtures and isotopic derivatization reagents (i.e., d_6_-Dns-Cl and d_6_-Dns-PP) were used. The ID-ISs and actual samples were prepared in parallel. At the end of the reaction, 5 μL of ID-ISs was added to the labeled samples. After vortex and centrifugation at 14 000 rpm for 10 min, the supernatant was submitted to the LC-MS analysis.

3. LC-MS conditions

Metabolite quantification was performed on a Shimadzu Nexera UPLC system interfaced with an 8060 triple quadruple mass spectrometer (Shimadzu, Kyoto, Japan) equipped with an electrospray ionization (ESI) source. The autosampler was kept at 4°C. The chromatography separation was performed on an Agilent Zorbax Eclipse XDB-C18 column (2.1 × 100 mm, 1.8 μm) at 50°C. Mobile phase A was 0.1% formic acid in the water, and mobile B was methanol. The chromatographic gradient was run at a flow rate of 0.40 mL/min as follows: for Dns-Cl derivatized products, 0-2 min (30% B), 2-5 min (30%-52% B), 5-15 min (52%-65% B), 15-20 min (65%-78% B), 20-23 min (78% B), 23-27 min (100% B), and 27-29 min (30% B); for Dns-PP derivatized products, 0-2 min (30% B), 2-5 min (30%-52% B), 5-15 min (52%-65% B), 15-20 min (65%-78% B), 20-22.5 min (78% B), 22.5-29 min (90% B), 29-38 min (100% B), and 38-40 min (30% B). The mass spectrometer was operated by scheduled multiple reaction monitoring (MRM) in positive ion mode with the optimal parameters as follows: spray voltage, 4.5 kV; nebulizing gas, 3 L/min; drying gas, 15 L/min; heat block temperature, 400 °C; desorption line temperature, 250°C.


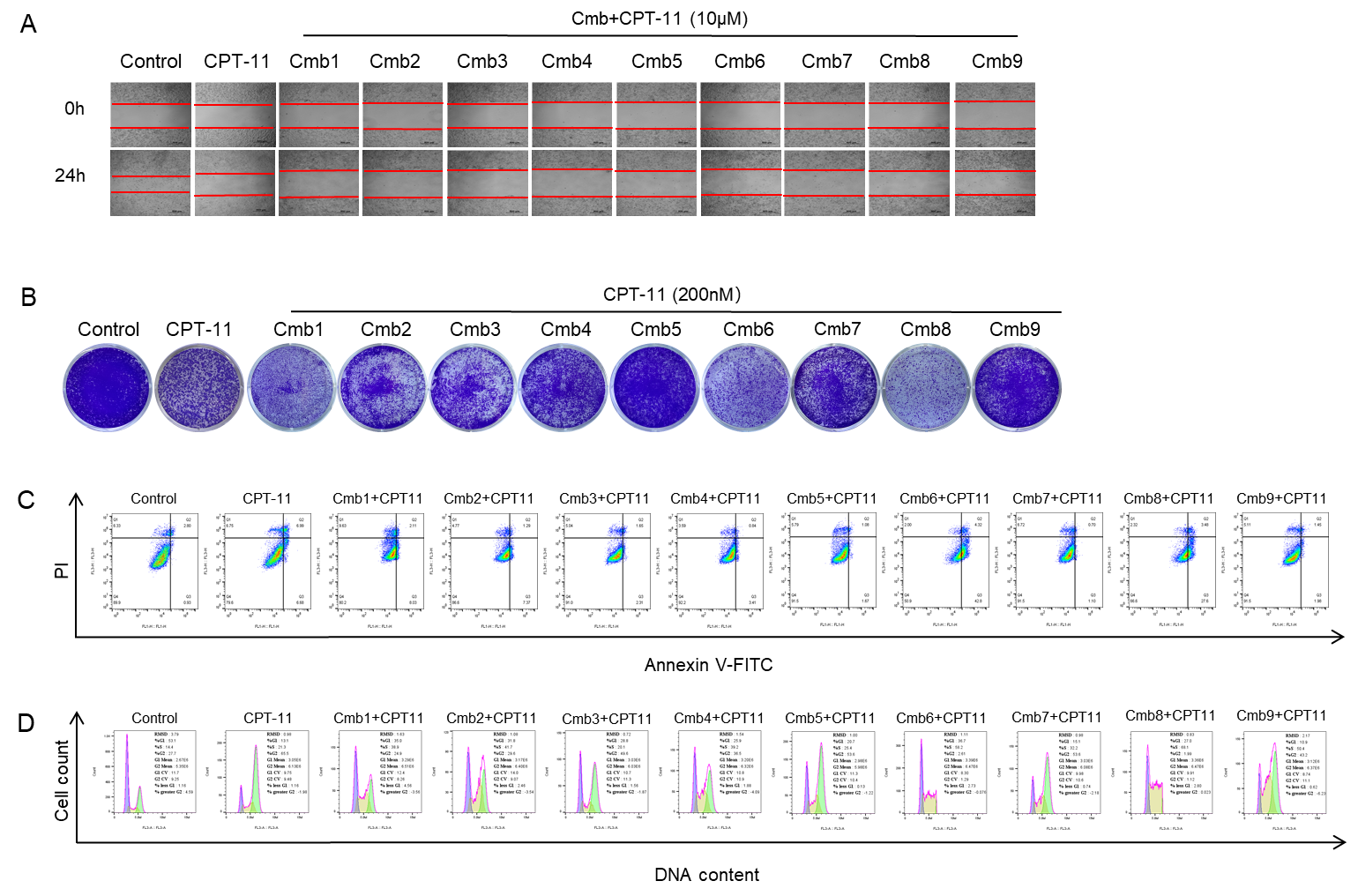


Figure S1. Data related to Figure 1. The original images of wound healing assay (A), clone formation experiment (B), apoptosis experiment (C), as well as cell cycle assay (D).


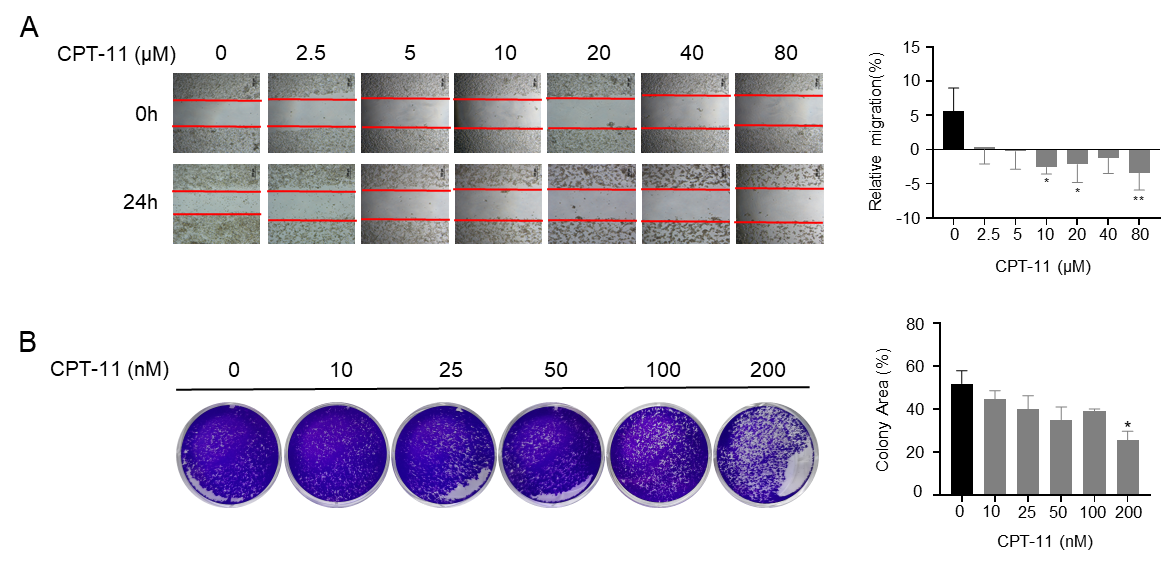


Figure S2. Cell migration assay (A) and cell colony formation assay (B) evaluating the anticancer effect of CPT-11 alone. One-way ANOVA with Tukey test, *p<0.05, **p<0.01, ***p<0.001, ****p<0.0001 in comparison with Control (no CPT-11).


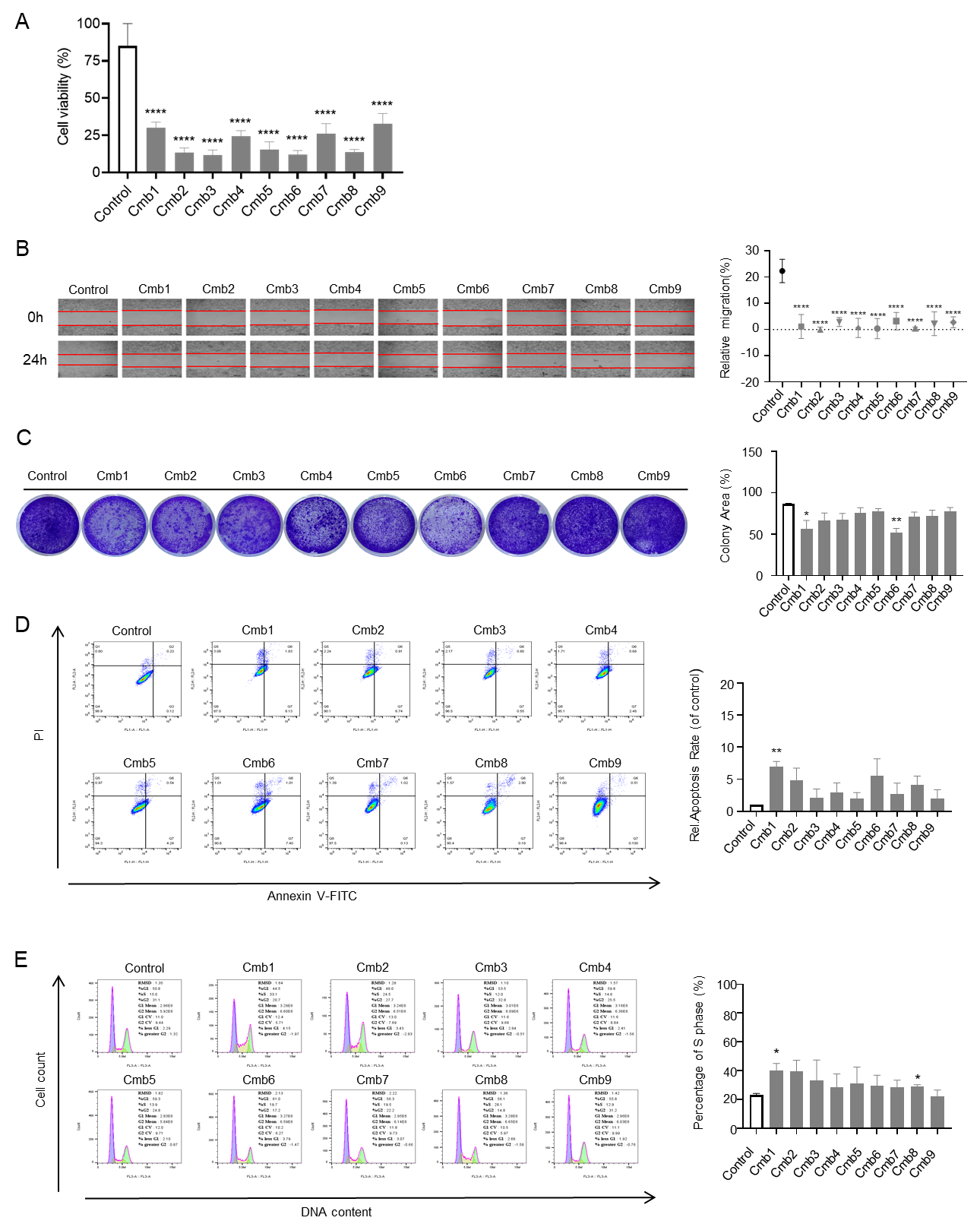


Figure S3. Cell viability assay (A), wound healing assay (B), clone formation experiment (C), apoptosis experiment (D), as well as cell cycle assay (E) of different combinations alone. One-way ANOVA with Tukey test, *p<0.05, **p<0.01, ***p<0.001, ****p<0.0001 in comparison with Control.


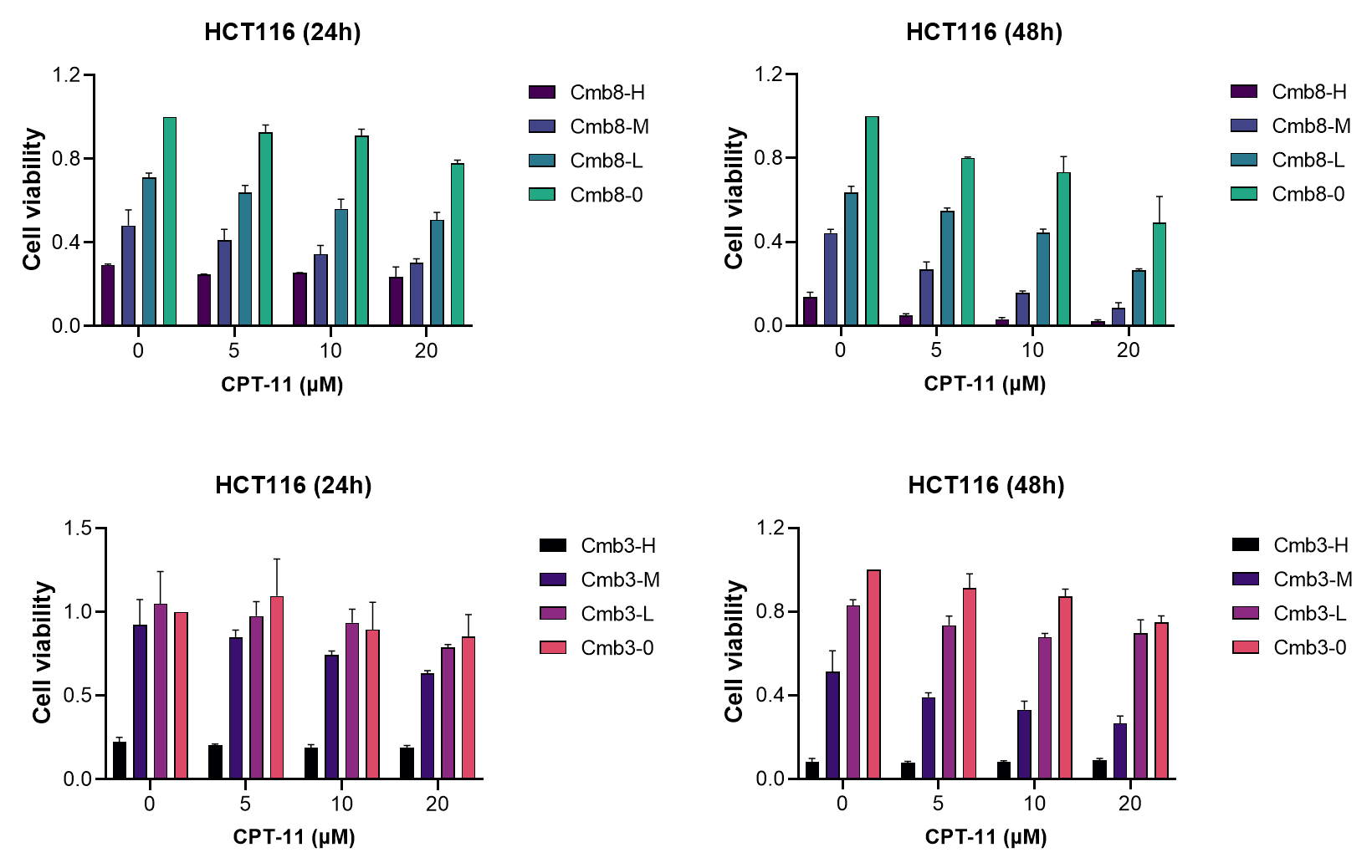


Figure S4. Cell viability assay at 24 h and 48 h on HCT116 cells evaluating the effect of Cmb8 or Cmb3 combined with CPT-11.


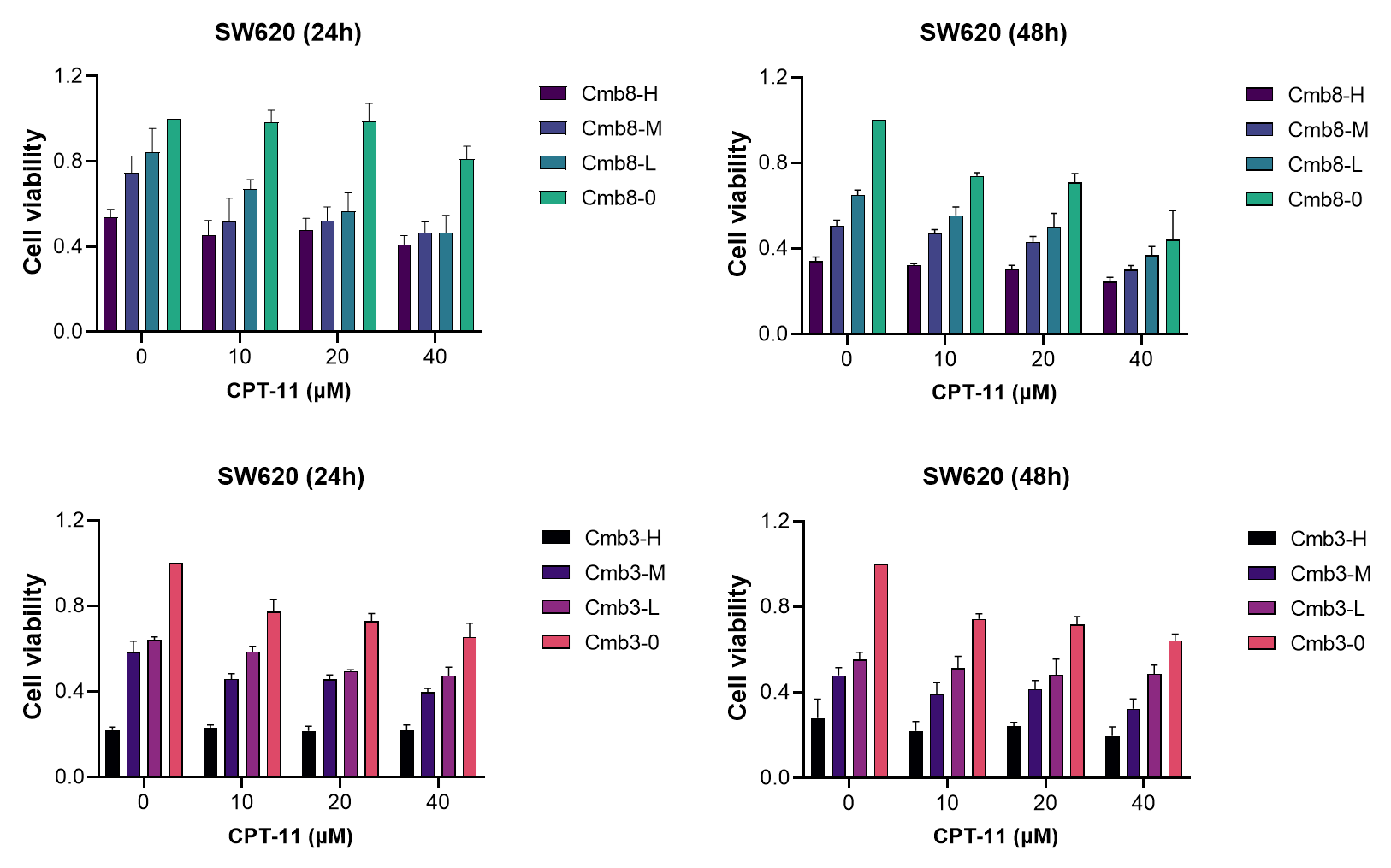


Figure S5. Cell viability assay at 24 h and 48 h on SW620 cells evaluating the effect of Cmb8 or Cmb3 combined with CPT-11.
